# ^177^Lu-PSMA radioligand therapy for isolated bilateral adrenal metastases from prostate cancer

**DOI:** 10.2340/1651-226X.2024.40017

**Published:** 2024-07-05

**Authors:** Elisabetta Perrone, Kriti Ghai, Aleksandr Eismant, Kornelia Konz, Richard P. Baum

**Affiliations:** a CURANOSTICUM Wiesbaden-Frankfurt, Center for Advanced Radiomolecular Precision Oncology, Wiesbaden, Germany; b Institute of Nuclear Medicine, Università Cattolica del Sacro Cuore, Rome, Italy; c Department of Endocrinology, Deutsche Klinik für Diagnostik, Wiesbaden, Germany

**Keywords:** Prostate cancer, adrenal metastases, ^68^Ga-PSMA PET/CT, ^177^Lu-PSMA radioligand therapy, theranostics

## Introduction

Prostate cancer is one of the most frequently diagnosed cancers in men worldwide [1]. The incidence of new cases has increased in recent years, primarily due to expanded screening recommendations and prostate-specific antigen (PSA) testing. However, the mortality rate remains relatively low compared to other cancer types [1], and it has shown a decreasing trend in recent years due to advancements in diagnosis and treatment. Ten-year survival rates for prostate cancer are generally high, reaching 98% in the USA [2]. Nonetheless, in cases of advanced disease, particularly in metastatic, hormone-resistant and chemotherapy-refractory tumors, the condition can be life-threatening and significantly affect the patient’s quality of life. When it comes to treatment options for prostate cancer, especially in situations where disease progression or recurrence is evident, and most or all available therapies have been exhausted, the use of prostate-specific membrane antigen (PSMA) labelled with the beta-emitting Lutetium-177 (^177^Lu) has emerged as a viable and safe choice. In recent years, numerous trials have explored the utility of ^177^Lu-PSMA therapy in patients with demonstrated PSMA-positivity via positron emission tomography/computed tomography (PET/CT). The VISION trial [3], for instance, randomized 831 metastatic castration-resistant prostate cancer (mCRPC) patients who had progressed post-taxane and post-androgen receptor pathway inhibitors (ARPI) to receive either ^177^Lu-PSMA-617 therapy plus supportive care or best supportive care alone. The primary outcomes assessed were overall survival (OS) and image-based progression-free survival (PFS). Results from the VISION trial demonstrated the efficacy of ^177^Lu-PSMA-617 therapy, with a significant improvement in OS and image-based PFS compared to the control group. Following these findings, regulatory approvals were granted in 2022 by the Food and Drug Administration (FDA) in the USA [4] and later by the European Medicines Agency (EMA) [5] for ^177^Lu-Vipivotide Tetraxetan, Pluvicto®. Moreover, the TheraP trial [6] compared ^177^Lu-PSMA-617 to chemotherapy with Cabazitaxel in 200 patients with mCRPC who had progressed after prior Docetaxel treatment. The primary endpoint assessed was PSA response, defined as a ≥50% reduction from baseline. Results from the TheraP trial revealed higher PSA response rates (66% vs. 37% in the Cabazitaxel group) and fewer grade 3/4 adverse events (33% vs. 53%) with ^177^Lu-PSMA-617 therapy, underscoring its potential as a treatment option for advanced prostate cancer patients who would otherwise require second-line chemotherapy. To explore the efficacy of PSMA radioligand therapy in an earlier clinical setting and mitigate chemotherapy-related adverse effects, the PSMAfore trial [7] enrolled 468 taxane-naїve mCRPC patients who had progressed after prior ARPI therapy. This prospective, randomized trial compared ^177^Lu-PSMA-617 to Abiraterone or Enzalutamide. Results presented at the 2023 congress of the European Society for Medical Oncology demonstrated that PSMA radioligand therapy prolonged radiological PFS (approximately 12 months vs. around 5 months in the ARPI change group) with a favorable safety profile.

In the context of metastatic disease, the most commonly affected sites are the bones, followed by lymph nodes [8]. It is worth noting that prostate cancer metastases to the adrenal glands are exceedingly rare, particularly when they occur in isolation (referred to as an oligometastatic pattern) [8]. Radium-223 dichloride (^223^Ra) is an alpha-emitting radiopharmaceutical that mimics calcium, binding to hydroxyapatite and forming complexes in bone sites with high turnover, such as skeletal metastases [9]. It was the first alpha-emitting agent approved by the FDA in 2013 (Xofigo®) for prostate cancer patients with painful bone metastases. In the ALSYMPCA trial [10], ^223^Ra proved effective not only in alleviating pain and delaying adverse skeletal events but also in extending OS. However, while bone-seeking agents like ^223^Ra are beneficial for skeletal metastases, they have limitations in treating metastatic disease with visceral involvement. In such cases, radioligand therapy targeting the membrane antigen PSMA, present on cancer cells regardless of metastatic sites, can be more effective.

## Case description

In May 2020, a 67-year-old patient received a diagnosis of prostate adenocarcinoma with a Gleason Score of 9 (4+5) and concurrent skeletal metastases in a disseminated pattern (high-volume disease, particularly in the spine and pelvis). At the time of diagnosis, the initial serum PSA value was 647 ng/mL. Treatment included androgen deprivation therapy (ADT) with a GnRH-analogue (Leuprorelin), six cycles of Docetaxel chemotherapy, and use of an androgen receptor inhibitor (Enzalutamide), resulting in remission of the disease. By October 2021, the PSA nadir had reached 0.5 ng/mL while under ADT with Leuprorelin/Enzalutamide. However, during follow-up, there was a serological (biochemical) recurrence, with serum PSA rising to 1.74 ng/mL in February 2022.

**Figure 1:**
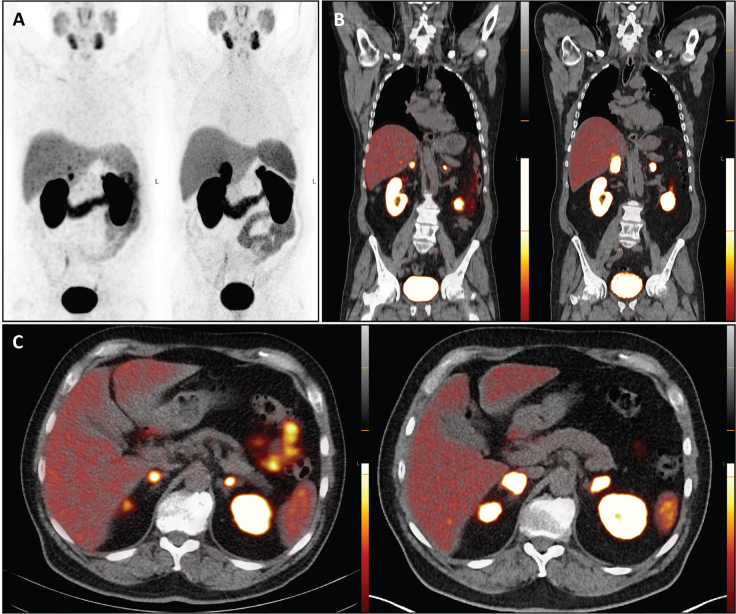
The PET/CT images with ^68^Ga-PSMA depict maximum intensity projection (A), coronal (B), and transversal (C) views. The images on the left are from February 2022, while those on the right are from March 2023. A visual comparison reveals disease progression in both adrenal glands over the 13-month period, despite ongoing androgen deprivation therapy. Specifically, in March 2023, there is increased PSMA uptake in the adrenal masses compared to February 2022. For instance, while the radiotracer uptake in the right adrenal gland appears bifocal and small in the February 2022 scan, a single intense and more extensive uptake is evident in the March 2023 scan. Notably, no new metastases or local recurrence were detected after this timeframe.

A restaging procedure was performed using PET/CT with PSMA labelled with Gallium-68 (^68^Ga-PSMA-11). The molecular imaging revealed disease progression characterized by isolated bilateral adrenal masses with intense PSMA-avidity in February 2022. Interestingly, there were no other sites with abnormal PSMA uptake; the previously known skeletal metastases appeared sclerosed on the co-registered CT, particularly visible in the spine and pelvis. ADT with Leuprorelin/Enzalutamide was continued, but approximately 1 year later, the PSA level increased to 8.2 ng/mL. A subsequent contrast-enanched CT and ^68^Ga-PSMA PET/CT in March 2023 confirmed further disease progression, with bilateral adrenal metastases increasing in size, and intensity of PSMA-uptake – the maximum standardized uptake values (SUV_max_) were significantly higher compared to the PET/CT from February 2022 (SUV_max_ 71.6 vs. 13.3 on the right; 42.2 vs. 13.9 on the left) (Figure 1A–C).

Given the refractory nature of the disease, ADT was discontinued, and a multidisciplinary team, in accordance with the EANM/SNMMI guidelines, confirmed the indication for ^177^Lu-PSMA radioligand therapy [11]. The management of adrenal metastases required a risk-benefit assessment, taking into account the rarity of adrenal insufficiency due to metastases, which typically requires the destruction of over 90% of adrenal tissue to cause glucocorticoid and mineralocorticoid deficiency [12]. On the other hand, bilateral adrenalectomy can be associated with significant debilitation and life-threatening complications like Addison’s crisis.

Although an attempt was made to biopsy the adrenal mass, it was technically unsuccessful and not repeated. At the time of treatment decision, the patient remained clinically asymptomatic, and adrenal tests showed normal serum cortisol and DHEA values, with only a slight elevation in ACTH. Given these factors, bilateral adrenalectomy was not performed, and the patient proceeded with ^177^Lu-PSMA radioligand therapy.

The patient underwent four cycles of ^177^Lu-PSMA therapy with a 6–8 weeks interval each (July, September, October, and December 2023), with a total administered activity of 32.9 GBq of ^177^Lu. For each cycle of therapy, a two-night stay in the hospital was planned. The therapy was well-tolerated, with no subjective acute or delayed adverse effects. Additionally, no hematological, renal, or hepatic toxicities were detected in laboratory exams conducted periodically throughout these months. Serum PSA values showed only a minimal reduction during the therapy cycles, decreasing from 29.1 ng/mL before the first treatment to 27.2 ng/mL after the last cycle.

**Figure 2:**
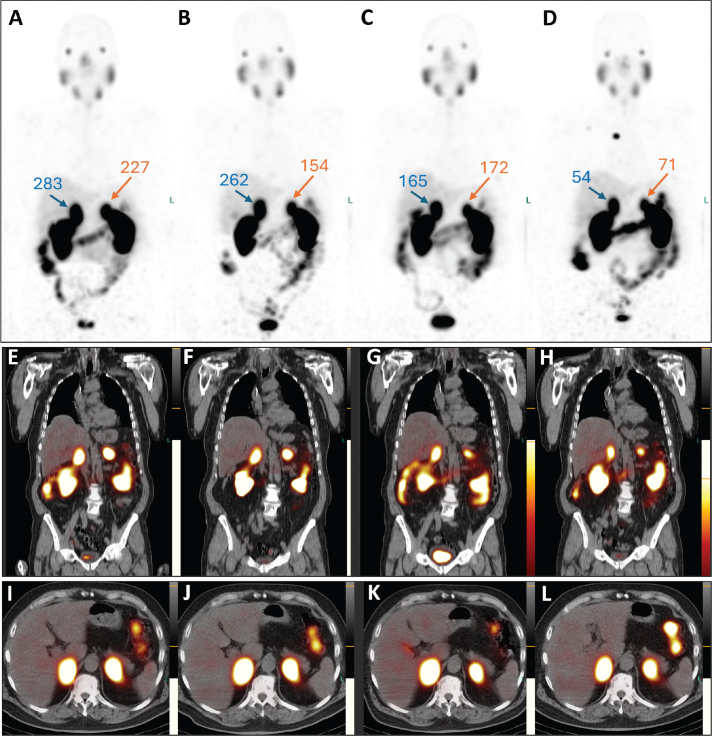
Post-therapeutic SPET/CT images. Maximum intensity projection images: A, July 2023; B, September 2023; C, October 2023; D, December 2023. Coronal: E, July 2023; F, September 2023; G, October 2023; H, December 2023. Transversal: I, July 2023; J, September 2023; K, October 2023; L, December 2023. Throughout the therapy cycles, bilateral adrenal masses show a decrease in both PSMA uptake and lesion size. The tumor-to-background ratio for each adrenal lesion, calculated based on the mean counts of radiotracer activity (as described in Figure 3), is provided.

**Figure 3:**
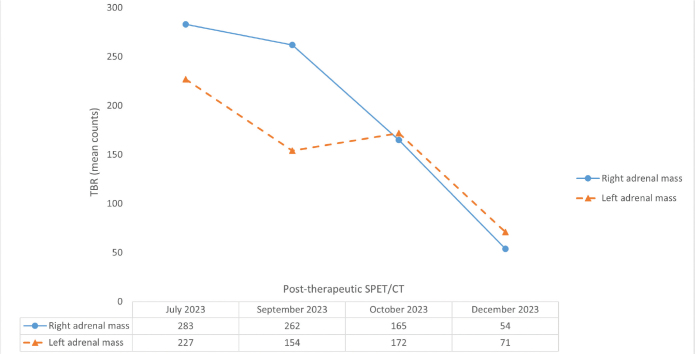
The upper part of the figure displays the trend of the tumor-to-background ratio (TBR) across the therapy cycles. These ratios were determined by considering the mean counts of radiotracer activity in both adrenal masses and in the gluteus muscle, the latter serving as the background reference. The lower part of the figure presents a table detailing the calculated TBR values after each therapy cycle. Overall, there was a decrease in TBR values (after four cycles of therapy: 81% reduction on the right and 69% reduction on the left), confirming partial remission of the disease. This semi-quantitative assessment is further supported by the observed reduction in adrenal metastases volume (50% on the right and 66% on the left).

Post-therapeutic ^177^Lu-PSMA single-photon emission tomography/computed tomography (SPET/CT) was performed after each treatment cycle. Visual (qualitative) evaluation revealed a decreasing uptake of the radiopharmaceutical in the bilateral adrenal metastases (Figure 2A–L). Furthermore, a semi-quantitative evaluation based on mean counts in both adrenal masses and background (using the gluteus muscle as reference) showed an 81% reduction on the right and a 69% reduction on the left after four cycles of therapy (Figure 3). Additionally, there was a reduction in the volume of the lesions between July and December 2023. Specifically, the mass in the right adrenal gland decreased from 5.2 × 3.1 x 6.0 cm to 4.9 × 2.2 × 4.5 cm (from 47.4 mL to 23.8 mL, a volume reduction of 49.7%), and the mass in the left adrenal gland decreased from 3.1 × 3.5 × 5.6 cm to 3.0 × 2.0 × 3.4 cm (from 29.7 mL to 10.0 mL; a volume reduction of 66.3%). Notably, no new distant metastases or local recurrence were detected. Therefore, after four cycles of radioligand therapy, partial remission of the disease was declared based on morpho-functional criteria observed on SPET/CT.

**Figure 4:**
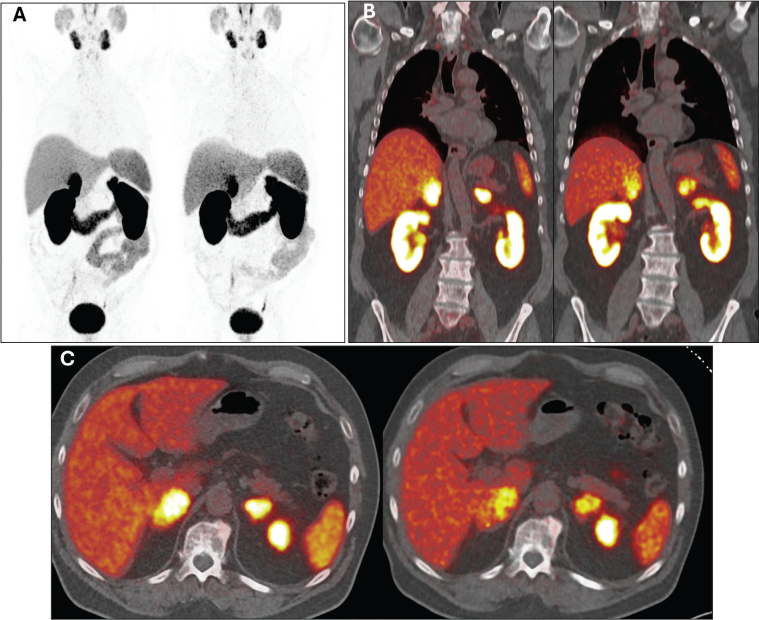
Maximum intensity projection (A), coronal (B), and transversal (C) images of ^68^Ga-PSMA PET/CT from March 2023 and March 2024 (the latter conducted during the latest follow-up) were obtained to reassess the disease burden and response to four cycles of ^177^Lu-PSMA therapy. In each section, imaging from March 2023 is displayed on the left, and from March 2024 on the right, illustrating notably reduced PSMA uptake by adrenal lesions. This indicates persistent partial response to therapy and ongoing disease control.

Partial remission of the metastases persists. In the latest follow-up in March 2024, 3 months after completing therapy, the patient remained in good health and reported normal activity levels. Lab results showed no clinically significant abnormalities, and PSA levels decreased to 19.7 ng/mL. A restaging ^68^Ga-PSMA PET/CT scan revealed continued therapy response, with further reduction in PSMA uptake by adrenal metastases, stable dimensions of adrenal lesions, and no signs of local recurrence or new distant metastases (Figure 4A–C).

This case report highlights the efficacy and safety of ^177^Lu-PSMA radioligand therapy in treating isolated bilateral adrenal metastases in an asymptomatic prostate cancer patient with preserved adrenal function, following disease progression under ADT with Leuprorelin/Enzalutamide.

## Discussion

The occurrence of isolated prostate cancer metastases to the adrenal glands is a rare phenomenon [8]. The biological basis of this involvement may be linked to the local steroid microenvironment, which is rich in androgen precursors capable of activating androgen receptor signalling, as demonstrated by immunohistochemistry on tumor tissues [13]. Additionally, in vitro testing has shown that both testosterone and cortisol can stimulate the growth of tumor cells [13]. While there have been a few reports of unilateral adrenal metastases detected by Fluorodeoxyglucose (^18^F-FDG) and ^68^Ga-PSMA PET/CT, some of which were successfully treated with adrenalectomy [14], and cases of detection through ^68^Ga-PSMA PET/CT due to biochemical recurrence after prostatectomy, managed with systemic treatment [15], isolated bilateral adrenal metastases remain relatively uncommon. These have been reported in patients with prostate cancer who were treated solely with hormone/chemotherapy [16, 17]. In addition to these reports, some authors have explored the utility of ^18^F-Fluciclovine PET/CT in detecting both unilateral and bilateral adrenal metastases [18, 19]. Recently, a case of radioligand therapy in a patient with adrenal involvement was documented [20]. The authors describe the case of a 66-year-old prostate cancer patient with bilateral adrenal metastases (oligometastatic pattern), progressed after Docetaxel, treated with four cycles of ^177^Lu-PSMA-617 (7.4 GBq each cycle). Treatment led to a significant PSA decrease and a near-complete response as shown by ^68^Ga-PSMA PET/CT; this case, similar to the patient presented in our report, did not manifest signs or symptoms of adrenal insufficiency and adrenal hormonal values remained in range during follow-up [20].

The case presented here focuses on the remarkable efficacy and safety of ^177^Lu-PSMA radioligand therapy in an asymptomatic patient with isolated bilateral adrenal involvement resulting from chemotherapy-refractory and hormone-resistant prostate cancer. The patient achieved partial remission of the disease (currently persisting) after undergoing four cycles of therapy and, notably, suffered from no adverse effects. Furthermore, the patient did not report experiencing xerostomia or a reduction in tear production. In fact, salivary and lacrimal glands are among the healthy organs that show high PSMA uptake and are exposed to radiation as a result of accumulation of the radiotherapeutic molecule. This can lead to radiation-mediated toxicity to the glands, reducing their normal secretory function, although this is rare with ^177^Lu-PSMA and more common when administering Actinium-225 (^225^Ac)-PSMA (an alpha-emitter), if no measures are taken to protect salivary glands [21].

It is important to emphasize that response to radioligand therapy is not always reflected in serum PSA levels. In the case described here, serum PSA values showed only a minor and non-significant decrease, yet the adrenal metastases exhibited a significant reduction in size, intensity, and the extent of PSMA uptake. This underscores the importance of considering multiple indicators when evaluating the effectiveness of such treatments.

Finally, the utilization of both pre-treatment ^68^Ga-PSMA PET/CT and post-treatment ^177^Lu-PSMA SPET/CT represents an excellent example of the practical application of radiotheranostics. This concept represents one of the most promising fields in radiomolecular (nuclear) medicine, as it seamlessly integrates both diagnostic and therapeutic aspects, offering a comprehensive approach to managing complex cases like the one presented in this report.

## Data Availability

Due to the nature of this work (case report), the data are not publicly available because they contain personal information that could compromise the privacy of the patient.

## Prior presentation

Contents of the present manuscript have not been previously published and have not been submitted elsewhere.
